# Active Control System to Prevent Malfunctioning Caused by the Pressure Difference in Gasket Plate Heat Exchangers Applied in the Oil and Gas Industry

**DOI:** 10.3390/s22124422

**Published:** 2022-06-11

**Authors:** Thiago Martins, Anderson Wedderhoff Spengler, Jorge Luiz Goes Oliveira, Kleber Vieira de Paiva, Laio Oriel Seman

**Affiliations:** 1 Graduate Program in Electrical Engineering, Federal University of Santa Catarina, Florianópolis 88040-900, SC, Brazil; 2 T2F, Thermal Fluid Flow Group, Federal University of Santa Catarina, Joinville 89219-600, SC, Brazil; anderson.s@t2f.ufsc.br (A.W.S.); jorge.goes@t2f.ufsc.br (J.L.G.O.); kleber.paiva@t2f.ufsc.br (K.V.d.P.); 3 Graduate Program in Applied Computer Science, University of Vale do Itajaí (UNIVALI), Itajaí 88302-901, SC, Brazil; laio@univali.br

**Keywords:** malfunctioning prevention, gasket plate heat exchanger, oil and gas industry

## Abstract

In the oil and gas industry, heat exchangers are subject to loads that cause malfunctioning. These loads are divided into thermal and mechanical stresses; however, most efforts are focused on studying thermal stresses. The present work reduces mechanical stresses by mitigating pressure events in a gasket plate heat exchanger (GPHE). GPHE requires that the hot and cold stream branches have approximately the same pressure. Thus, the work focuses on controlling the pressure difference between the branches. A test bench was used to emulate, on a small scale, the typical pressure events of an oil production plant. A control valve was used in different positions to evaluate the controller. In the experiments, it was observed that the best option to control the pressure difference is to use a hydraulic pump and control valve in the flow of the controlled thermal fluid branch. The reduction in pressure events was approximately 50%. Actuator efforts are also reduced in this configuration.

## 1. Introduction

Heat exchangers are devices that facilitate the heat transfer between two fluids without mixing the flows [1]. The device is used in different applications that require heat transfer, and, for this reason, there are different types of heat exchanger designs [2]. The oil and gas industry can have configurations with sealed plates, printed circuits, shells, and tubes. A widely used heat exchanger is the plate heat exchanger (PHE) [3]; they are suitable for liquid-to-liquid heat exchange applications as long as the hot and cold flow branches have approximately the same pressure [4].

When analyzing the process, it is generally convenient to operate and control the exchanger through an overall heat transfer coefficient [5]. Fakheri [6] presented a new methodology for network analysis of series-connected heat exchangers and provided expressions for determining the size and heat transfer rate in individual heat exchangers in a network. Thus, studies on heat exchangers are commonly developed to control the thermal load. Recent studies are still looking for alternatives in this sense, mostly including intelligent controls. Jin et al. [7] conducted experimental research on heat exchanger control based on hybrid identification of time and frequency domain. A controller based on artificial intelligence is presented in [8], where the authors use a fuzzy logic controller capable of adequately stabilizing the temperature of the heat exchangers. Soesanti and Syahputra [9] followed the same line of control by fuzzy logic through simulations. Another work uses the fuzzy controller in conjunction with the feed-forward–feedback method involved in a PID controller. Recently, Tugashova and Zatonskiy [10] reviewed methods to improve the control of the heat exchanger. Other lines of research focus on the damage caused to exchangers through temperature variations.

The simultaneous action of a corrosive environment and cyclic stresses induce the device to fatigue failure. The two major causes of fatigue are thermal overload and mechanical overload [11]. The control of thermal variables is focused on improving the heat exchange efficiency and increasing the system life expectancy. An example in the objective was to identify geometries that provide acceptable performance over a desired operational period considering fouling in heat exchangers used in crude oil preheating trains [12]. In Trafczynski et al. [13], the objective was to increase the system’s energy efficiency and minimize greenhouse gas emissions through a linear control system of the parallel flows of a heat exchanger network. Arsenyeva et al. [14] presented the developments in the design theory of PHEs, aiming to improve heat recovery and energy use efficiency. Another example of thermal optimization is the extensive network of preheating heat exchangers to reuse as much energy as possible from a refinery distillation column [15].

Some refineries and other industries use compact plate heat exchangers for this task. Picón and Rumbo [16] presented PHEs in energy recovery systems to reduce the number of units and the potential benefits in renovation projects. The approach for multifluid cases is demonstrated in a crude oil preheat train containing 12 heat exchangers. An exchanger efficiency study aimed at applications in the dairy industry was presented in [17]. Mathematical optimization for PHEs applied to the ammonia production industry in [18]. Alam and Kim [19] proposed a graphical method and rules to integrate the heat exchanger network, considering the pressure variation in the distillation columns. The work relates the problem with pressure variation, but the proposed method sought to optimize the plant for energy savings. In this way, the network structure of exchangers and their operation are optimized mainly for efficiency. It is also necessary to control mechanical cyclic loads and mechanical overloads for the same reason.

Heat exchangers are thoroughly studied for failure prevention using the maintenance of the inactive control of static and dynamic loads (e.g., fatigue). Techniques observe variations at critical points and suggest design-level solutions to increase device life. Zhao et al. [20] evaluated fatigue life in a high-pressure exchanger in coal-fired power plants during transient processes of operational flexibility regulation. The authors presented data guidelines for the safety and maintenance of coal-fired power plants during these operational flexibility regulatory processes. Panjeshahi and Tahouni [21] presented a classic case study of premature failures in heat exchangers and described how to predict device life. Other approaches study the failure modes of devices and suggest solutions in design and/or preventive maintenance. Addepalli et al. [22] presented a study that evaluates degradation mechanisms in heat exchangers to assess new requirements for future designs of heat exchangers. The work [23] addresses corrosion in heat exchange devices in general. Rezaei et al. [24] analyzed the corrosion failure of heat exchanger tubes used in a petrochemical refinery. Both works end by presenting corrosion prevention methods. Finally, a comprehensive review of the common causes of failure in heat exchangers is presented in [25].

Rydén [26] reported the leading causes of the failure of GPHEs in an oil production plant. The information presented was obtained through interviews and questionnaires with professionals who work in maintaining these devices. Reports point out that mechanical fatigue causes friction and fluid mixing and can be caused by either continuous pressure pulsations in the system or frequent starts/stops causing pressure variations. Pressure shocks and continuous pressure variations are mentioned as reasons for the rupture of GPHE gaskets. Moreover, several cases are reported where there have been recurrent gasket burnout failures because the corrective action only includes the new gasket and not the elimination of pressure pikes. Severe and recurring failures often occur because of a failure to identify the source of the problem, which could be outside the GPHE. The main faults due to pressure variations caused by oil well oscillations are induced by gasket rupture caused by pressure shocks in the system. In this case, the GPHE must be opened and the gasket replaced. Internal leaks (fluid mixing) are caused by fatigue due to continuous pressure variations. The corrective action is to replace the damaged plates.

Recent studies have been increasingly concerned with the event of variation in the pressure difference between branches of GPHEs. The failure mode studies presented by Pelliccione et al. [27] concluded that failures were due to mechanical fatigue. This fact was evidenced by the observed characteristics (striations, marks, nucleation of multiple cracks, and propagation of transgranular cracks). In the case study presented, the GPHE is designed to cool fresh water (temperature—32 °C; pressure—0.6 to 0.8 MPa) using seawater (temperature—29 °C; pressure—0.3 to 0.4 MPa). Martins et al. [28] reported that the pressurization of a single branch (a difference of 10 bar between branches) significantly impacts the mechanical request. The equivalent von Mises stress is up to 80% lower than in double load tests in single tests. Martins et al. [28] show that the relationship between pressure and stress is practically linear. The GPHE used showed a stress increase of approximately 32 MPa (Von Mises) for a pressure increase of 2 bar in one of the branches. The stress was obtained in the critical region of the plate distribution area. This result quantifies the stress imposed during the pressurization event of a single branch (increase in the pressure difference between branches) and is directly related to the solution addressed in the present work.

However, recent literature does not include malfunction prevention using active pressure control. In this sense, this work presents novelty by investigating active pressure control to minimize the causes of heat exchanger malfunctions. The purpose is to demonstrate that it is possible to perform this task through a simple active controller that actuates the variations in the pressure difference between branches of a GPHE. The problem was presented by Petrobras S.A., which reported malfunction by events of pressure variations in plate heat exchangers. The pressure events were quantified by Martins et al. [28], and the results show that the high-pressure difference is more harmful than equivalent high pressures in both branches. To emulate these events, a reduced scale of a typical profile pressure variation in oil gas plant production was used. A test bench was used to perform the variations in amplitude and frequency to emulate the oil production branch using the control of the hydraulic pump. A control valve was positioned in different configurations for the control, and another pump was controlled to provide flow water on the second branch. The configurations obtained were compared to the system without the control to investigate the performance of the active control in the mitigation of pressure events.

## 2. Contextualization

### Oil and Gas Industry

The history of fluctuations in exchanger inlet conditions (i.e., pressure, temperature, flow) in the oil and gas industry is significant. Neglecting these variations results in severe system malfunctioning. Malfunctioning can be highlighted, such as gasket expulsion (which represents about 45% of malfunction occurrences), friction wear of gaskets and plates, erosion, deformation due to pressure blows, fatigue, and corrosion, among others [26]. The consequences of inadequate control are also observed through corrective maintenance (standard in these applications) performed on heat exchangers. The result is a significant financial loss in system maintenance and downtime.

Controlling these quantities by multiphase flows can be complicated. Multiphase flows make it difficult to predict the thermal load while promoting high-input oscillations that result in thermomechanical stresses in the heat exchanger and other plant components. Thus, the present work simplifies the problem by adopting single-phase flows only.

The loads acting on the exchanger components arise in mechanical and thermal stresses. Fatigue occurs with cyclical variations of these loads. In this way, it is possible to increase the life of the heat exchanger by reducing the amplitude and frequency of the load cycle. According to ASME Sec, the range of equivalent mechanical stresses and the thermal loads in each cycle form the set of parameters necessary to obtain the effective alternating load equation [11]. When mechanical stresses are reduced, the effective alternating stresses decrease. Alternating stress is used in conjunction with material characteristics to describe the rate of damage per load cycle; this determines the life of the heat exchanger. Malfunctioning must be minimized in amplitude and frequency by controlling system pressures.

**Figure 1:**
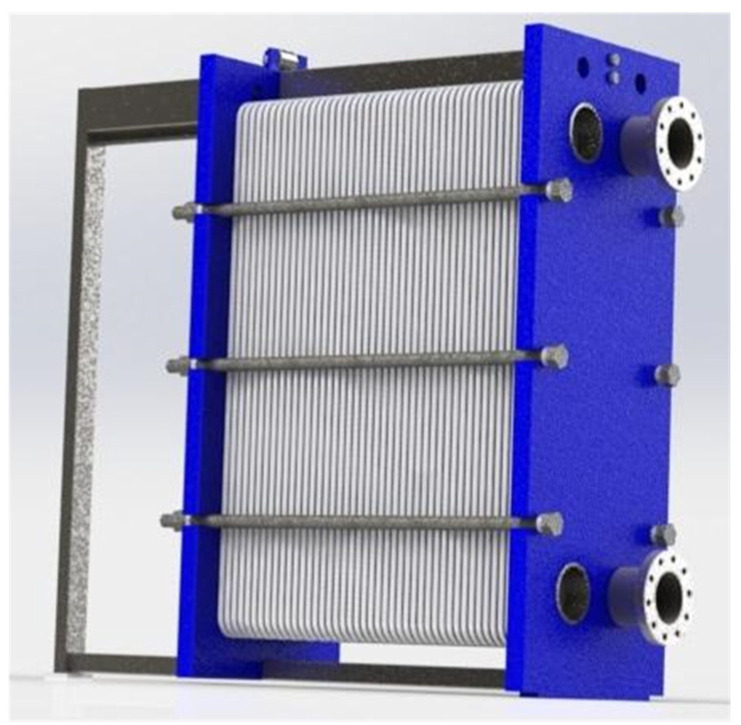
Gasket plate heat exchanger (GPHE) used in pressure control tests.

There are different types of plate heat exchangers, and each one has its particularities in terms of the pressure and temperature levels supported. Here, we consider a gasket plate heat exchanger model (Figure 1). The maximum stresses on GPHEs are limited due to plates and gaskets. Gaskets impose restrictions on operating temperatures (maximum 160—250 °C) and pressures (up to operating pressure 25—30 bar) [2]. The nature of the fluids that can be handled must also be considered in a GPHE design. An advantage of the GPHE is that this model can be dismantled for cleaning and maintenance (necessary in applications with solid waste and severe fouling conditions). For GPHEs, the magnitude of the pressure difference between the branches is one of the principal demands. Pressure surges observed in the oil well branch generate oscillations in this pressure difference and, consequently, premature fatigue. The other branch is responsible for warming the flux produced through the warm water branch in closed-loop thermal control.

To reduce the oscillation amplitudes in the pressure difference, it is necessary to control the flux of both branches. The oil well branch (OWB) is responsible for the oscillation, and the controller can attenuate disturbances. The branches’ control can be implemented through the flow rates in the thermal bath branch, represented in this paper by the water branch (WB). Thus, the parameter to be controlled is the variation in the ratio of the pressure difference between the branches (Pressure Difference Variance—PDV), which can be described:

PDV = (∆P_OWB_ − ∆P_WB_) + P_0_, (1)

where ∆P_OWB_ is the pressure variation at the OWB, and ∆P_WB_ is the pressure variation at the WB. Ideally, ∆P_OWB_ − ∆P_WB_ = 0, where P_0_ is the design pressure difference.

## 3. Materials and Methods

### 3.1. Plates Geometry

**Figure 2:**
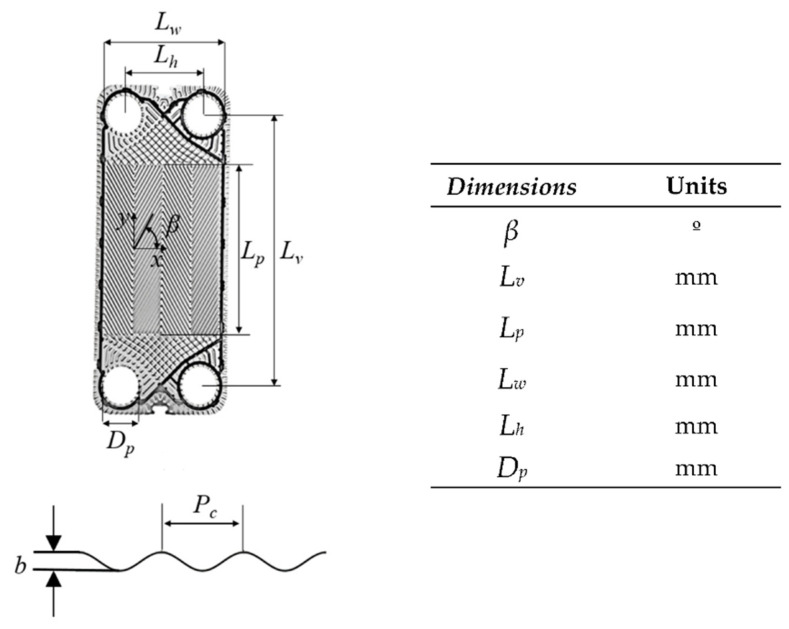
Main dimensions of the plates.

The plates’ geometries, dimensions, and corrugations vary according to the design of the heat exchanger manufacturers, established by their industrial application. Figure 2 illustrates the overall dimension of the plates employed in this work.

### 3.2. Test Bench

**Figure 3:**
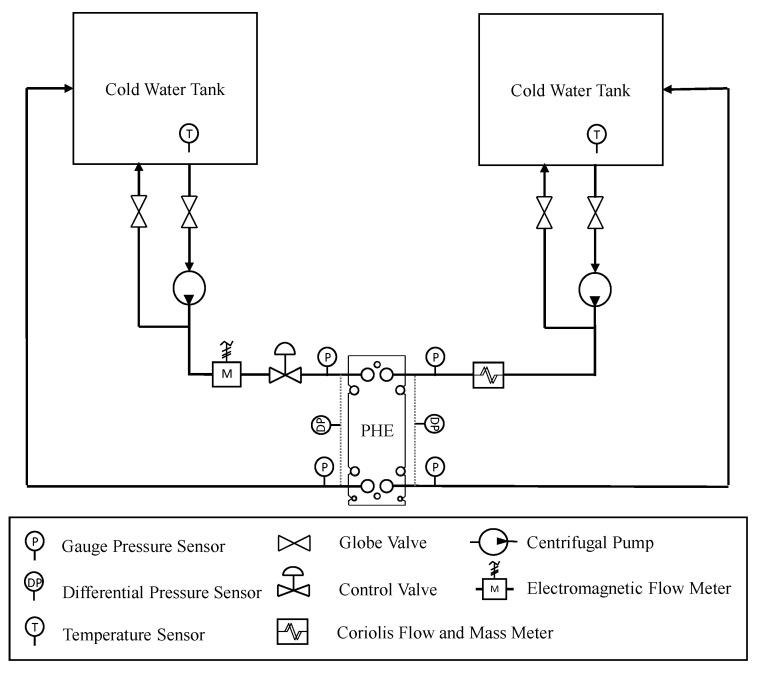
Test bench schematic.

The experiment was conducted on a test bench composed of two water tanks, two frequency inverters with hydraulic pumps, and a control valve. The apparatus also has pressure, temperature, and flow sensors for monitoring the process. The schematic is shown in Figure 3.

Hydraulic pumps have their limits imposed by frequency inverters that serve to control and generate profiles of system disturbances. The control valve had its position changed between the branches during the tests. Two models of pressure sensors are used; one makes a manometric reading, and the other reads the pressure difference at the inlets and outlets of the heat exchanger branches. Flow and temperature sensors were not used in the process control. The main components that make up a workbench are shown in Table 1 and Table 2.

Two pressure sensors are used—a model for reading gauge pressure and another for reading the differential pressure of the inlets and outlets of the heat exchanger channels. The differential pressure sensor (PX409-0150W) has its operating limit at 15 psi (1 bar). The gauge pressure sensor (PX409-100GI) supports pressures up to 100 psi (6.9 bar). The transducer’s outlet is a current signal from 4 mA to 2 mA and can be supplied with voltages of up to 10 V. The temperature sensors were used only to monitor the system and were not used to obtain the results of this work due to the imposed simplifications.

To measure the flow in each branch of the bench, two flow sensor models are included. For one of the branches, an electromagnetic sensor (Rosemount 8700 M) is used. A Coriolis sensor (Emerson CFM 200) measures mass flow for the second branch. Both provide a current output of 4 mA to 20 mA.

**Figure 4:**
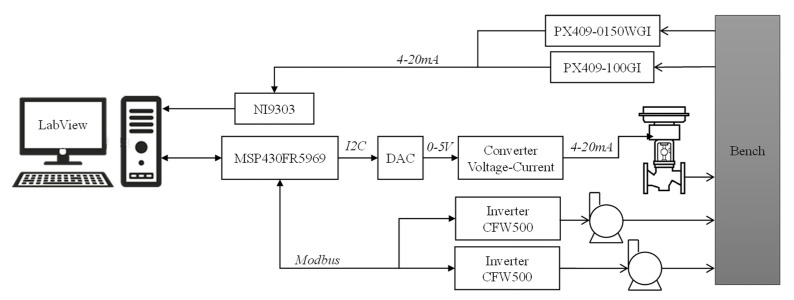
Control system. Relationship between components and their communications.

In addition to sensors and actuators, the test bench has several components for control, monitoring, and data transmission, as shown in Figure 4. The microcontroller used to act on the inverters and the control valve is the MSP430FR5969 model. This device is responsible for RS485/Modbus communication with inverters (38,400 bit/s), UART for the computer (115,200 bit/s), and I2C to act on the control valve (100 Kbit/s). The microcontroller receives a command from the interface and transmits it via the I2C interface to a digital-to-analog converter that generates a 0–5 V voltage signal to act on the control valve. This signal then passes through another converter, a voltage-to-current converter, which generates a 4–20 mA current signal. In the case of pressure sensors, the generated signals are acquired by the NI9203. The main electronic components are shown in Table 3.

The microcontroller operation routine starts with an interruption via UART through communication with the computer. The other protocols are executed through the pooling method; that is, it sends the command and waits for the actuator’s response to continue the operation (for inverters only).

The system is controlled and monitored through LabView software. The LabView program was designed using six operating states: initialization, read, processing, command, save data, and termination. The system’s default operating cycle is determined by only four of the six states (excludes initialization and termination states).

### 3.3. Operating Time

The total time of an operating cycle is approximately 174.02 ms. This time involves the entire period necessary for the system to read the sensors and the inverter status, process the data, act on the pumps, act on the valve, and finally save the data. The communication times of the subsystems were obtained considering the microcontroller’s communication rates and operating frequency. Table 4 presents an overview of the times.

The quantity (Qty) in Table 4 refers to the number of times communication occurs in a single interface operation cycle. The control valve depends on I2C communication. Pump control requires a drive response for each branch. The reading is carried out in two steps (read command, inverter response). The monitored data from the inverter are the pump frequency and operating status.

The valve response time depends on the UART serial communication between PC and microcontroller, on the I2C communication time between microcontroller and converter, and the accommodation time of the valve itself. Communication time is approximately 276 µs. The response time of the valve positioner and actuator will depend on the valve’s internal pressure. To a 10% move command and a flow rate of 1.34 LPS (standard for the valve and positioner described), the valve reached the target after 2.619 s. The following times are considered to act on the hydraulic pump: UART serial communication between the PC and the microcontroller, microcontroller with the frequency inverter, and pump acceleration. In this case, communication takes place in approximately 182 µs. The drive starts the pump acceleration or deceleration ramp at 5 Hz/s (factory default acceleration) after the processing and communication times. For a 5 Hz shift command, Pump 1 takes approximately 1.057 s to initiate the speed shift. Pump 2, for the same input condition, takes approximately the same time 1.083 s. Actuator response times are shown in Table 5.

The data presented in the third column of Table 5 show the time it takes for the system to observe a change in flow after sending a command to the actuator. Therefore, this time includes issues such as processing information by LabView.

### 3.4. Test Configuration

One of the branches emulates the pressure events in OWB, and the second branch represents the WB. The aim is to apply the PDV between the branches of a GPHE. The actuators must compensate for the pressure variations imposed by the control and re-establish the desired nominal pressure difference between the branches. So, four configurations were chosen for the control system. Test 1 has a configuration where only the hydraulic pump acts on WB to compensate for the variations observed in the pressure difference. Test 2 used only the control valve in OWB to attenuate pressure variations directly in the disturbance induced by the hydraulic pump. Test 3 combined the two previous ones; the pressure difference must be re-established using the pump in WB and the valve in OWB. Finally, the test 4 configuration consists of the pump with the valve in WB to control PDV together.

Settings were tested for different pressure inputs in magnitude and frequency. Pressure variations in magnitude correspond to abrupt pressure changes in OWB using the maximum acceleration allowed by the frequency inverter (5 Hz/s). The setup values chosen for the task were 1 Hz, 2 Hz, 5 Hz, and 10 Hz, corresponding to pressure variations of 0.04 bar, 0.10 bar, 0.24 bar, and 0.50 bar, respectively. The maximum value was determined to avoid possible damage to the test bench and its components.

Pressure variations in the frequency correspond to cyclic loads. The pump’s inverter speed control is used to introduce acceleration and deceleration. The values chosen for the frequency input were 0.2 Hz/s, 0.5 Hz/s, and 1 Hz/s, corresponding to accelerations of 0.008 bar/s, 0.020 bar/s, and 0.038 bar/s, respectively. Although the tests were set up by pump acceleration, the interest value is the pressure variations’ frequency.

The maximum amplitude set at the inputs was kept at approximately 0.25 bar. The pump control in WB does not influence the controller’s response to frequency variations; for this reason, only one of the tests was verified in frequency compensation. The control valve, when not used, performs the restriction task to induce the pressure difference between the branches (approx. 50%). This application is evidenced in test 1 and characterizes relationships between the oil and water branches commonly observed in the application. This restriction can be associated with fouling or just the difference between the loads associated with each branch. The test conditions described in this section are shown in Table 6.

**Figure 5:**
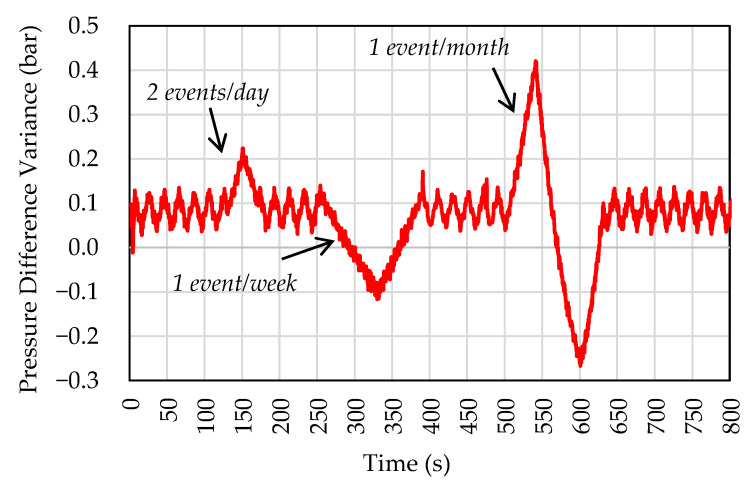
Disturbance to test the maintenance control of the pressure difference between branches.

In addition to the magnitude and frequency tests, a third perturbation mode was used. This test emulates the pressurization events of a typical oil production plant, with reduced amplitude variations in scale. In this way, the acceleration of the disturbance was reduced, but the frequency was maintained. Figure 5 shows the small-scale perturbation used in our tests. Figure 4 also shows the frequency of each event in an actual plant. The desired nominal pressure difference between the branches was around 0.1 bar. Pressure variations were introduced by changing the speed of the pump connected to the branch representing OWB.

### 3.5. Uncertainty Analysis

Uncertainties propagations were obtained through repeated testing of the controller. The uncertainty was of 0.19 bar and differential pressure of 0.02 bar for the gauge pressure transducer. The electromagnetic sensor (Rosemount 8700M) has an uncertainty of ±0.5%. For the second branch, a Coriolis sensor (Emerson CFM 200) measures mass flow with an uncertainty of ±0.1%.

## 4. Results

### 4.1. Test 1—Pump in Water Branch

The first tests intended to keep the minimum pressure difference constant between the branches by acting on the frequency inverter allocated in WB. The pressure variation was entered into the OWB branch. The controller actuated on the pump frequency inverter, which is in the branch of WB. In this experiment, it was considered that the pressure difference between the branches should be zero, PDV = 0.

**Figure 6:**
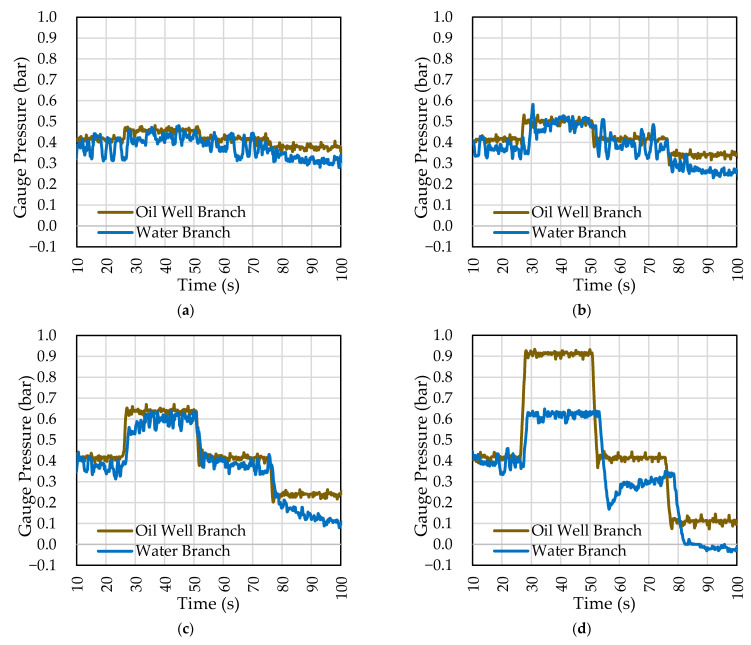
Manometric pressure of the branches for entry in varied amplitude. Pump performance in the WB. Pressure variation applied to the OWB in: (**a**) 0.04 bar; (**b**) 0.10 bar; (**c**) 0.24 bar; (**d**) 0.50 bar.

Figure 6 shows the gauge pressures for each branch for abrupt pressure variations in OWB. The controller in WB achieves a satisfactory response, making the pressure in WB close to that of OWB. In the case of Figure 6d, with an amplitude variation of 0.5 bar, the objective was not achieved. It should be noticed that the control valve allocated to branch WB (in this test) induces a restriction of 50% to the diameter of the pipe. This fact directly implies the hydraulic pump’s capacity of pressure gain compensation.

Table 7 compiles the main results obtained. The first column shows the hydraulic pump setup frequency (Hz) with the respective pressure variation in OWB. The settling time equals the time required for the PDV value to reach approximately 5% of the final value in each case. Settling times were obtained by the average of the three variations of each case. In the case of the variation in 10 Hz, the value settling time obtained does not represent a controller period but a time to limit the achieving pump. So, this caused the settlement time to be disregarded. It is important to remember that controller efficiency, in this paper, is mainly associated with stationary error and overshot.

The variance is the dispersion mean showing the distance of the means of the steady-state data. Steady-state error is the offset of the setpoint (a PDV equal to zero) after the PDV has stabilized. Maximum overshot is the difference between the steady-state and the highest peak pressure observed for a single variation. In this case, the largest overshot values were observed when reducing the gauge pressure on the OWB. The last column shows the hydraulic pump efforts (minimum and maximum frequencies).

A PID with high proportional gain was used for the tests performed to respond to the highest-pressure differences in a shorter time. The response has a high overshot as well as a longer oscillatory response. The higher the step applied, the greater the overshot due to the gain. The most significant error occurred with setup at 10 Hz, where the actuator reached its speed limit. The values obtained in this response time come from the pressure drop, where the system has more difficulty re-establishing the desired pressure. In the event of positive pressure peaks, the controller can respond quickly. These values can be adjusted by changing the variables of the PID controller.

**Figure 7:**
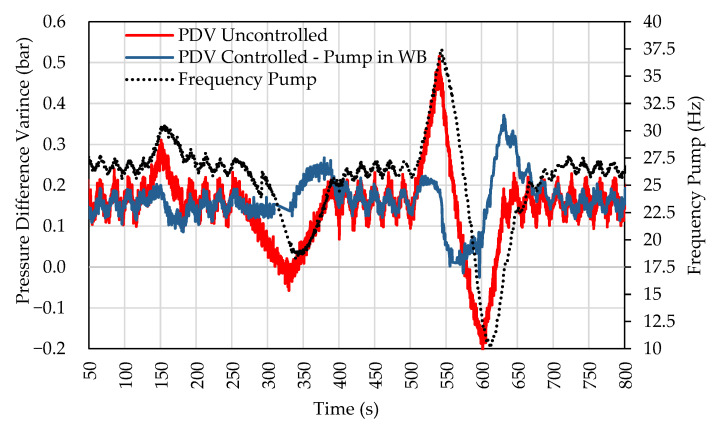
Response to system disturbance. Pump performance in the WB.

After obtaining the pressure input in amplitude results, the test was performed with the real perturbation on a reduced scale. Figure 7 compares the uncontrolled PDV and the pump-controlled PDV in the WB. The setpoint value was PDV = 0.15 bar.

The controller follows the pressure value set at 0.15 bar. The pressure difference for the setpoint was close to –0.15 bar and 0.2 bar during the most critical points of the tests performed, showing a significant reduction in these points. The standard deviation around the point of interest reduced from 0.085 bar to 0.047 bar. Figure 6 also shows the pump frequency in WB and how the actuator operated to smooth out pressure events in PDV. For higher peaks, the pump operated close to its operating limits.

### 4.2. Test 2—Valve in Oil Well Branch

The second test aimed to reduce pressure peaks in OWB. For this, the control valve was used as an actuator in OWB. Table 8 shows the results obtained for inputs with different amplitudes.

The maximum response time is obtained when a pressure drop occurs. For cases where the pressure variation was more significant than 0.24 bar, the operating limits of the valve were quickly reached. The PDV does not reach the setpoint in these cases. The actuator takes longer to compensate for pressure drops than for pressure build-up. The valve could attenuate pressure peaks and practically linearize variations below 0.05 bar.

**Figure 8:**
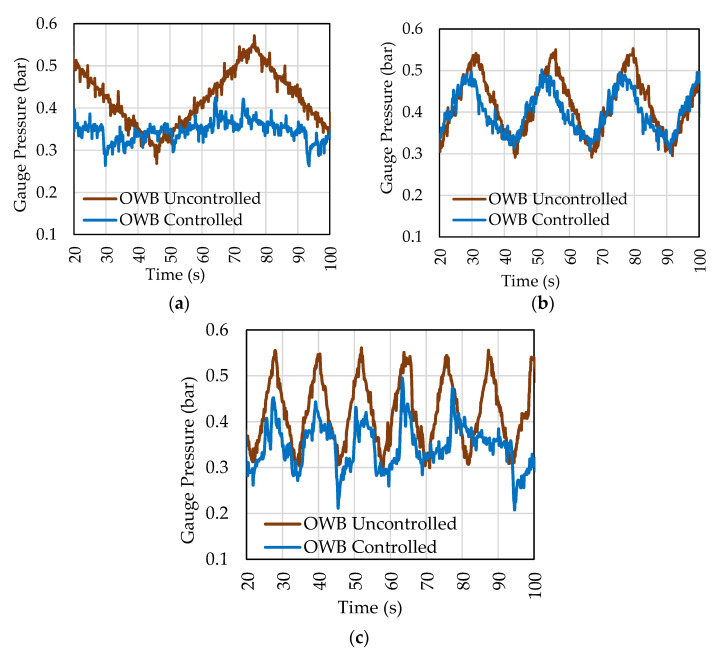
Variation in frequency of the pressure applied in OWB. Values described as pump acceleration, pressure acceleration, frequency of variations: (**a**) 0.2 Hz/s = 0.008 bar/s = 0.017 Hz; (**b**) 0.5 Hz/s = 0.020 bar/s = 0.042 Hz e (**c**) 1.0 Hz/s = 0.038 bar/s = 0.084 Hz.

After obtaining the configuration response for the amplitude input, tests were performed by varying the acceleration of pressurization events. Responses to inputs with different accelerations are shown in Figure 8. The tests have the same amplitude but different frequencies. The pressure acceleration was reduced until the controller could completely mitigate pressure variations. The control valve was able to eliminate variations in pressure difference if the acceleration was less than 0.008 bar/s, and the valve was operating within its limits. Acceleration is equivalent to a slew rate of 0.5 bar/min. Amplitude and cyclic variation are considered, and the maximum frequency the controller can express is 0.017 Hz (1/58 Hz).

**Figure 9:**
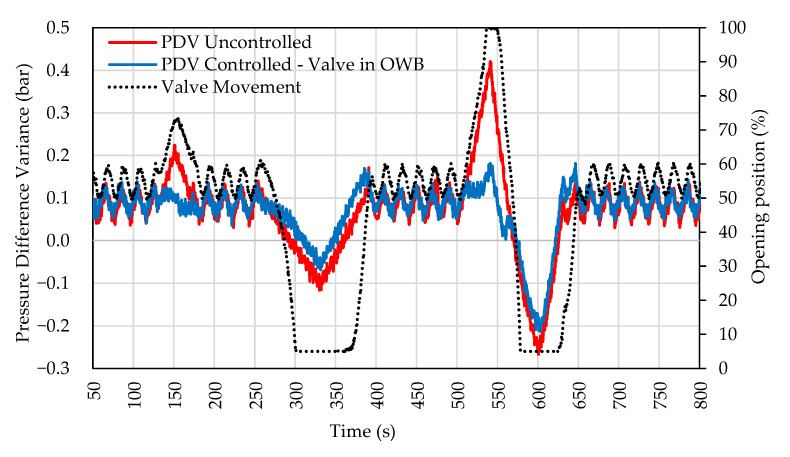
Response to system disturbance. Valve actuation in the OWB.

Figure 9 shows the comparison of the uncontrolled PDV with the PDV controlled by the valve in OWB. The graph shows that the valve is suitable for reducing positive PDV peaks by opening the control valve in OWB. However, the valve control did not significantly improve for negative peaks (where the pressure in WB is greater than OWB) even when it reached the lower operating limit. The graph also shows the movement of the valve that has the initial opening at 50%.

The highest frequency in the disturbance was 0.05 Hz with an amplitude of 1 bar, that is, an acceleration of 0.2 bar/s. In this case, the controller could not smooth out the variations in pressure difference because the frequency result is analogous to the case in Figure 8b.

### 4.3. Test 3—Pump in Water Branch and Valve in Oil Well Branch

Using the two previous configurations simultaneously, it was possible to obtain better results. The purpose of this test was to include the advantages of each configuration in the same test. The results show that the stationary error and overshot values are improved in exchange for the response time, as shown in Table 9.

The valve controller was set to a higher gain to improve the PDV control. The valve operated within its limits range at 0.24 bar and 0.5 bar configurations. In these cases, the response time increased. The pump only reached its limit at the 0.5 bar setting. Thus, the choice of this configuration caused the pump to respond slowly, generating a longer time for stability. On the other hand, at 0.04 bar and 0.10 bar settings, the error scale, overshot, and response time were lower than in tests 1 and 2.

**Figure 10:**
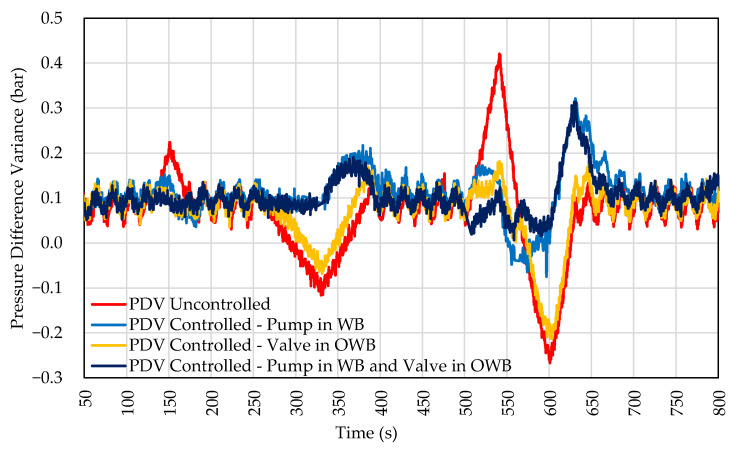
Response to PDV in amplitude in the OWB. Control by valve in OWB and pump in WB.

In Figure 10, the comparison between tests 1, 2, and 3 is presented. As can be seen, the simultaneous control of the pump in WB and the valve in OWB showed improvements, mainly in amplitude variation rates.

All settings showed an improvement in the control compared to the uncontrolled curve (uncontrolled PDV). The control using only the pump could attenuate positive and negative peaks of the pressure difference between the branches. In the case of valve-only control, only positive pressure spikes were attenuated, and the configuration can mitigate pressure variations at low frequency. In the configuration that uses both solutions, the positive and negative peaks were attenuated, but an improvement in amplitude control was observed compared to the solution using only the pump in WB.

### 4.4. Test 4—Pump and Valve in Water Branch

Actuators reached their limits when a high-pressure variation was introduced in all previous tests. To reduce the efforts of the pump on the WB, the control valve was attached to the WB. With this change, there was an improvement concerning the controller, and the actuators’ efforts were significant. Table 10 shows the result obtained using the pump and valve configuration in WB.

Response time, steady-state, and overshot decreased significantly compared to previous tests. The controller used in the valve has a higher gain concerning the inverter controller. This option made the valve responsible for most of the actuation in control. Both actuators had their actuation reduced in this experiment.

**Figure 11:**
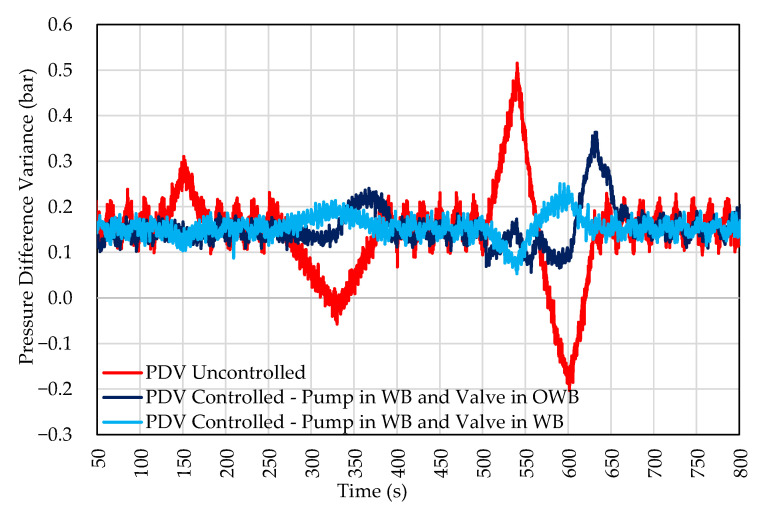
Response to system disturbance. Valve and pump actuation in the WB.

The magnitude of pressure variations for the 0.04 and 0.10 bar inputs was negligible in this configuration. The pressure difference between the branches was attenuated, and the actuators never reached their range limits at the same instant. The behavior is presented in the PDV control chart for the input with the disturbance scale shown in Figure 11.

Figure 11 shows a significant improvement in POS control compared to the configuration presented in test 3. This configuration allowed controlling inputs with high amplitudes (10 Hz—0.50 bar). In Figure 11, it is also possible to observe that the high-frequency disturbance was also slightly attenuated.

**Figure 12:**
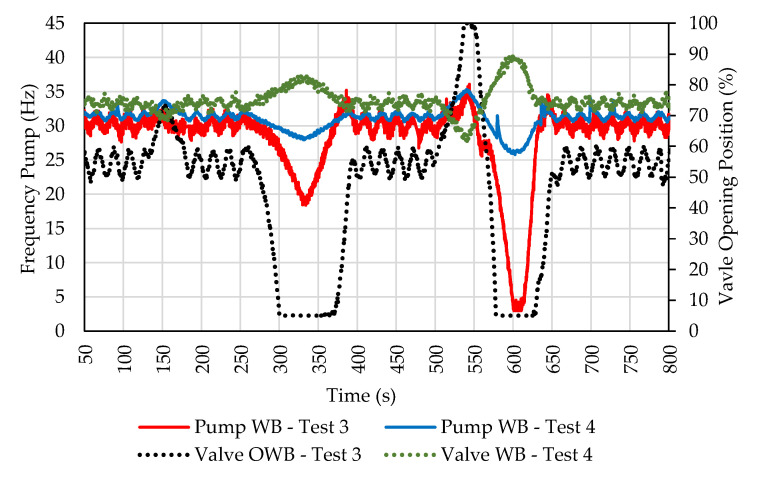
Comparative graph of the efforts of the actuators in tests 3 and 4.

Another advantage of this setup is the reduction in actuator efforts. The comparison of actuator efforts in tests 3 and 4 is shown in Figure 12. In test configuration 3, the control valve limits are quickly reached. However, in test 4, they are not. When compared to the previous setting, it is possible to observe the inversion of displacement in 325 and 600 s. With the help of the valve, the pump does not need to reduce its speed to control the pressure drastically.

## 5. Discussion and Conclusions

This work proposes the mitigation of malfunctions in gasket plate heat exchangers using the control of pressure events common in oil plants. The approach aimed to control the variation in the pressure difference between branches of a plate heat exchanger. The main conclusions are listed below:

- The system re-establishes the condition of an initial pressure difference for pressure variations below 0.25 bar in a maximum time of 5.65 s, 8.26 s, and 4.21 s in tests 1, 2, 3, and 4, respectively. In test 2, the valve reached its operational limit. In variations above 0.25 bar, only tests 3 and 4 successfully re-established the pressure difference in 10.74 s and 9.49 s, respectively;
- For variations in frequency, the valve could linearize the pressure at frequencies below 0.017 Hz. The standard frequencies in the actual plant are in the order of 0.04 Hz. This frequency is represented in full scale by the maximum acceleration of 0.15 bar/s. The amplitudes are equivalent to 2 bar. On the other hand, the points of greatest acceleration, around 0.5 bar/s, have low frequencies and can be controlled.
- Based on the background presented, the best results among the tested configurations were observed in test 4. Martins et al. [28] described the mechanical stress as having a ratio of 32 MPa for a difference of 2 bar between the exchanger branches. In this configuration, we reduced the pressure difference by 50% in events with greater pressure differences.
- The test 4 configuration was still able to minimize the efforts of the actuators. The valve performed the full opening movement to relieve pressure when the pump accelerated abruptly to compensate for the pressure gains of the opposite branch.

The authors believe that the best solution presented can be improved by including a control valve in the oil well branch. The results show that the valve in the oil well branch can mitigate slow pressure variations. For this, in future research, the use of oil is suggested to evaluate the control of valves in oil wells to mitigate the pressure difference in GPHEs.

## Figures and Tables

**Table 1:** Actuators of the test bench.

Component	Model	Range/Limits	Communication/Command	Step
Pump/Inverter	3 HP WEG/CFW500	3–60 Hz	RS485/Modbus	0.1 Hz
Valve/Positioner	ARI-STEVI Smart 440/441/NT-1000L	0–100% (block completely open)	4–20 mA	0.5%

**Table 2:** Sensors of the test bench.

Component	Model	Range	Communication	Accuracy
Gauge Pressure Sensor	PX409-100GI	0–6.9 bar	4–20 mA	0.5% FS
Differential Pressure Sensor	PX409-0150W	0–1.0 bar	4–20 mA	0.5% FS
Temperature Sensor	RTD PT100-PMA-1/8-6-1/8-R-3	−100–400 °C	-	0.15 + 0.002 °C
Electromagnetic Flow Meter	Rosemount 8711SHE	0.01–12 m/s	4–20 mA	0.25%
Coriolis Flow and Mass Meter	Emerson CFM200M418	0–0.024 m^3^/s	4–20 mA	±0.10%

**Table 3:** Main electronic components.

Component	Features and Operation
Computer	Data processing, controller, interface, and result storage. i5-6200U 2.3 GHz, 8 GB de RAM.
Microcontroller MSP430FR5969	Communication with frequency inverter and control valve. UART-interrupt; Modbus pooling.
Digital-to-Analog Converter	Communication with microcontroller I2C, 12 bit resolution, output 0 to 5 V.
Voltage-to-Current Converter	Input 0 to 5 V, output 4 mA to 20 mA.
NI9203	16 bit resolution, inputs –20 mA to 20 mA, sample 200 kS/s.

**Table 4:** Electronic control system times.

Component	Qty.	Proc ^1^	UART	I2C	Modbus	Total
Control Valve	1	10 µs	26 µs	240 µs	-	276 µs
Control Pump	2	<1 ns	26 µs	-	156 µs	182 µs
Read Inverter	4	20 µs	52 µs	-	312 µs	384 µs
Read Pressure	100	1 ms ^2^	-	-	-	100 ms
Interface and Process	1	174.02 ms ^3^	-	-	-	174.02 ms

^1^ The processing time involved in the task; that is, the response time of the converters or processing in subsystems. ^2^ Rate of 1 KHz continues, with 100 samples on average. ^3^ Approximate operating time of the interface.

**Table 5:** Flow stabilization time in the system after the command is sent to the actuators.

Set Command	Communication Time	Actuator Step	Actuator Response
Valve (10%)	276 µs	1.34 LPS	2.619 s
Pump 1 (5 Hz)	182 µs	5 Hz/s	1.057 s
Pump 2 (5 Hz)	182 µs	5 Hz/s	1.083 s

**Table 6:** Matrix of configurations of the tests performed.

	Pump Branch	Valve Branch	Pressure Variation
Test 1	WB	-	Magnitude and disturbance at scale
Test 2	-	OWB	Magnitude, frequency, and disturbance at scale
Test 3	WB	OWB	Magnitude, frequency, and disturbance at scale
Test 4	WB	WB	Magnitude and disturbance at scale

**Table 7:** Quantitative response of pressure difference control for various amplitude inputs. Acting by frequency inverter in WB.

Setup	Settling Time	Variance	Steady-State Error	Overshot Max.	Pump Frequency
	(s)	(Bar)	(Bar)	(Bar)	(Hz)
1.0 Hz—0.04 bar	2.640	0.039	0.08	0.10	27.9–34.8
2.0 Hz—0.10 bar	4.352	0.037	0.08	0.17	24.7–37.3
5.0 Hz—0.24 bar	5.646	0.035	0.15	0.28	15.5–40.0
10.0 Hz—0.50 bar	- ^1^	0.064	0.28	0.44	3.0–40.0

^1^ Related to the limitations of the actuators. The lower and upper limits of the pump (3 Hz and 40 Hz).

**Table 8:** Quantitative response of the pressure difference control for various amplitude inputs with actuation of the control valve in OWB.

Setup	Settling Time	Variance	Stationary Error	Valve Opening
	(s)	(Bar)	(Bar)	(Bar)
1.0 Hz—0.04 bar	7.017	0.001	0.025	56–73
2.0 Hz—0.10 bar	9.973	0.010	0.095	45–80
5.0 Hz—0.24 bar	- ^1^	0.015	0.100	3–100 ^2^
10.0 Hz—0.50 bar	- ^1^	0.012	0.270	3–100 ^2^

^1^ The upper and lower limits of valve movement were reached in this test. ^2^ Lower limit determined at 3% valve opening.

**Table 9:** Response of the pressure difference control for various amplitude inputs, with the actuation of the control valve in OWB and the pump in WB together.

Setup	Response Time	Stationary Error	Overshot Max.	Valve Movement	Pump Frequency
	(s)	(Bar)	(Bar)	(%)	(Hz)
1.0 Hz—0.04 bar	<3 ^1^	0.039	- ^2^	56–73	24.5–28.6
2.0 Hz—0.10 bar	<3 ^1^	0.035	0.074	43–82	23.6–29.3
5.0 Hz—0.24 bar	8.261	0.031	0.179	3–100	17.3–34.5
10.0 Hz—0.50 bar	10.743	0.071	0.232	3–100	3.0–40.0

^1^ Response time less than 3 s. ^2^ Overshot state error less than 0.02 bar.

**Table 10:** Quantitative response of the PDV control in amplitude. Control by valve and pump in WB.

Setup	Response Time	Stationary Error	Overshot Max.	Valve Movement	Pump Frequency
	(s)	(Bar)	(Bar)	(%)	(Hz)
1.0 Hz—0.04 bar	- ^1^	- ^1^	- ^1^	58.0–67.0	18.0–19.0
2.0 Hz—0.10 bar	- ^1^	0.014	- ^1^	57.7–67.9	17.7–19.3
5.0 Hz—0.24 bar	4.213	0.029	0.075	53.2–100	18.2–22.0
10.0 Hz—0.50 bar	9.490	0.048	0.097	45.4–100	17.0–22.2

^1^ Amplitude of data in the same order of magnitude as the variance of the data. Settling time cannot be determined (σ^2^ = 0.012).
